# Factors influencing recurrent varicose vein formation after radiofrequency thermal ablation for truncal reflux performed in two high-volume venous centers

**DOI:** 10.1016/j.jvsv.2023.08.014

**Published:** 2023-09-11

**Authors:** Domenico Baccellieri, Vincenzo Ardita, Alfonso Pannone, Ferdinando.B.A. Valente, Rosalba Lembo, Roberto Chiesa, Germano Melissano

**Affiliations:** a Department of Vascular Surgery, San Raffaele Scientific Institute, Vita-Salute University School of Medicine, Milan, Italy; b Department of Vascular Surgery, Azienda Ospedaliera San Camillo-Forlanini, Rome, Italy; c Department of Anesthesia and Intensive Care, IRCCS San Raffaele Scientific Institute, Milan, Italy

**Keywords:** Accessory saphenous vein, Great saphenous vein, Radiofrequency thermal ablation, Varicose vein recurrence

## Abstract

**Objective:**

Recanalization of the saphenous vein trunk after endovenous radiofrequency ablation (RFA) is often associated with recurrent varicose veins (RVVs) or recanalization. This study aimed to assess the long-term results of RFA of the great saphenous vein (GSV) and identify the risk factors for GSV recanalization and RVVs during follow-up for patients presenting to dedicated outpatient vein centers.

**Methods:**

All consecutive patients with incompetent GSVs who underwent RFA between 2009 and 2019 were retrospectively analyzed. The primary study end points were freedom from GSV recanalization and the RVV rate during follow-up. The secondary study end points were the postoperative complication rate and the risk factors for GSV recanalization and RVVs. Univariate and multivariate analyses were performed to identify the potential risk factors for GSV recanalization and RVVs.

**Results:**

During the study period, 1568 limbs were treated in 1300 consecutive patients (mean age, 53.5 ± 12.9 years; 71.9% women; CEAP [clinical, etiology, anatomy, pathophysiology] C2-C6; venous clinical severity score >5). Technical success was achieved in 99.7% of cases. At a mean follow-up of 57.2 ± 25.4 months, the GSV occlusion and freedom from reintervention rates were 100% and 100% within 1 week, 97% and 95.7% at 1 year, 95.2% and 93.1% at 3 years, and 92.4% and 92.8% at 5 years, respectively. The recurrence rate was 10% (n = 158) during the follow-up period. On multivariate analysis, a direct confluence of the accessory saphenous vein into the saphenofemoral junction (odds ratio [OR], 1.561; 95% confidence interval [CI], 1.0-7.04; *P* = .032), a history of pregnancy >2 (OR, 3.68; 95% CI, 1.19-11.36; *P* = .023), C4 (OR, 6.41; 95% CI, 1.36-30.28; *P* = .019), and preoperative GSV diameter >10 mm (OR, 1.82; 95% CI, 1.65-4.03; *P* = .043) were risk factors for GSV recanalization. Moreover, age >70 years (OR, 1.04; 95% CI, 1.01-1.06; *P* = .014) and incompetent perforator veins (OR, 1.17; 95% CI, 0.65-2.03; *P* = .018) were also risk factors for RVVs.

**Conclusions:**

RFA is a safe technique to ablate the GSV with a low complication rate and durability during 5 years of follow-up. However, patients with a high clinical score and those with direct confluence of the accessory saphenous vein into the saphenofemoral junction experienced higher long-term GSV recanalization and RVV rates.

**Article Highlights**

• **Type of Research:** A multicenter, retrospective, nonrandomized cohort study
• **Key Findings:** Radiofrequency ablation of the great saphenous vein resulted in a high occlusion rate during follow-up, with a recurrence rate of 10% and improved patient quality of life.
• **Take Home Message:** Radiofrequency ablation of the great saphenous vein is feasible and safe, with a high occlusion rate and a low rate of complications in the medium and long term.

In the past two decades, radiofrequency thermal ablation (RFA) has been recognized as a safe and effective minimally invasive treatment option for patients with chronic vein disease (CVD) secondary to great saphenous vein (GSV) reflux. Studies have shown that RFA results in similar occlusion rates compared with the traditional open technique, with occlusion rates of 95% for RFA and 96% for open repair.^1^ RFA offers advantages compared with open repair, such as avoiding the need for general anesthesia, fewer complications, and improvement in patient quality of life, leading the European Society for Vascular and Endovascular Surgery guidelines to consider it as the first-choice treatment.^2^

Despite the success of RFA, mid- and long-term recurrence has been reported with a variable incidence rate ranging from 7% to 32%, similar to the recurrence rates observed after open repair (GSV high ligation and stripping [HLS]).^3^ This suggests that uncertainties still exist regarding the factors contributing to GSV recanalization and recurrent varicose veins (RVVs) after RFA. Identifying patients at higher risk for GSV recanalization and RVVs is crucial for improving long-term treatment outcomes.

The literature contains several studies exploring potential risk factors for long-term RVVs and GSV recanalization after RFA; however, the reported data on this topic has continued to be debated.^4^^,^^5^ The present study aims to address this knowledge gap by assessing the long-term results for the GSV occlusion rate and the need for reintervention for patients with incompetent GSVs who underwent RFA. Furthermore, the study seeks to evaluate the risk factors associated with GSV recanalization and the onset of RVVs after RFA. The present research aims to provide valuable insights into improving the long-term success of RFA as a treatment option for CVD secondary to GSV reflux.

## Methods

### Study design

This is a retrospective and nonrandomized study performed at two high-volume venous centers: San Raffaele Hospital and San Camillo Forlanini Hospital. All participants exhibited CVD grade C2 to C6 using the CEAP (clinical, etiology, anatomy, pathophysiology; C2, varicose vein with symptoms; C3, swollen ankle; C4, skin changes; C5, healed leg ulcer; C6, active ulcer) classification and underwent RFA of the GSV with the ClosureFast catheter (VNUS Medical Technologies; Covidien) in accordance with the instructions for use.^6^ The collected data were derived from a prospectively maintained computerized database and included details on preoperative, intraoperative, and postoperative data.

Preoperatively, color Doppler ultrasound (CDUS) was used to evaluate GSV incompetence and the saphenofemoral junction (SFJ), with estimation of the external (adventitia-to-adventitia) and internal (intima-to-intima) diameters of the GSV and characteristics of outflow and reflux. The retrograde flow was identified at the SFJ and at three points along the thigh and/or leg, with reflux >0.5 second and a GSV diameter >4 mm indicating eligibility for treatment.^2^ The Valsalva technique was used to measure and assess the degree of reflux in the thigh area. The compression technique was used to determine the existence of superficial and/or deep reflux in the leg. The presence of an accessory saphenous vein (ASV) was also identified.

The inclusion criteria for RFA were determined from the clinical and CDUS standard recommendations outlined in the international guidelines.^2^ The exclusion criteria for RFA were severe tortuosity, a GSV diameter >20 mm, a distance between the skin and GSV of <0.5 cm, SFJ dilatation >2 cm, GSV duplication, a history of deep vein thrombosis (DVT), pregnancy and lactation, active cancer, symptomatic peripheral arterial disease, and severe systemic disease. Furthermore, patients with an incompetent small saphenous vein and femoral vein were excluded from treatment preoperatively.

The principles of the Declaration of Helsinki were followed throughout the study. All patients provided written informed consent on the standard consent form provided by our institution for the anonymous collection of data for the CE-approved device.

### Follow-up examinations

All patients underwent clinical and CDUS examinations within 1 week and 1, 3, and 5 years after treatment. In accordance with the report by Dermody et al,^7^ complications were evaluated and assessed during the initial postoperative visit and at 1 week postoperatively. These examinations aimed to discern patients with recurrence (RVV with or without GSV recanalization) and to determine the severity of any symptoms. Diagnostic and follow-up CDUS scans were performed by the same practitioners.

RVVs were defined as the reopening or persistence of varicose veins after previous RFA of the GSV, with or without phlebectomy or foam sclerotherapy, as reported in the European Society for Vascular and Endovascular Surgery guidelines.^2^ CDUS examinations were conducted to determine the perioperative technical success and treatment efficacy and to detect postoperative GSV occlusion, the necessity for reintervention, and the durability of RFA.

Technical success was defined as the capacity to deliver the ablation catheter to the targeted vein from the access point and deploy radiofrequency energy for the entire length of the incompetent vein, totally ablating the insufficient saphenous trunk. Treatment efficacy was defined as the absence of vein recanalization after the procedure. GSV obliteration was defined as the lack of blood flow in a not totally compressible GSV tract from 3 cm distal to the SFJ along the entire length of the treated vein. Recanalization was characterized as the presence of blood flow in ≥5 cm of the targeted treated GSV segment, disregarding the presence or absence of reflux. Durability was specified as the absence of RVV and neovascularization (formation of new blood vessels in an abnormal tissue or position) in the groin assessed by CDUS examination at follow-up.

Two groups of patients were analyzed: those without recurrence (group A) and those with recurrence (group B). Among the patients with recurrence, three subgroups were identified: those with RVVs and GSV recanalization (group B1), those with RVVs without GSV recanalization (group B2), and those with GSV recanalization without RVVs (group B3).

### End points

The primary study end point was the recurrences rate, including GSV recanalization and RVV onset during follow-up at ≤1 week and 1, 3, and 5 years after intervention. The secondary study end points were the postoperative complication rate, including hyperpigmentation, pain, ecchymosis, paresthesia, phlebitis, endovenous heat-induced thrombosis, DVT, and pulmonary embolism ≤1 week from intervention, and the risk factors for GSV recanalization and RVVs. The clinical outcomes were also evaluated using the CEAP classification and venous clinical severity score (VCSS). Clinical improvement was considered significant when it had decreased by ≥30% compared with the baseline VCSS during follow-up.

### Procedure

Ablation of the GSV was performed aseptically in the operating theater under tumescent anesthesia (Klein solution), using a percutaneous, Doppler-guided approach.^8^ Highlights of these procedures have been previously described.^8^ Access to the vein was achieved distal to the dilated segment using a 20-guage microneedle under CDUS guidance without the need for local anesthesia to prevent venous spasm. A 0.018-in. guidewire was then inserted into the needle, at which point, local anesthesia was administered at the puncture site and a 7F radial introducer sheath (Radiofocus; Terumo) was placed. Next, radiofrequency catheters were inserted through the sheath and advanced 2 cm below the SFJ under ultrasound guidance. Tumescent anesthesia was then applied around the saphenous vein, with ultrasound guidance and a roller pump fitted with a 22-guage, 3.5-in. spinal needle and an assembled infusion set. Thermal ablation was then performed under compression of the ultrasound probe, using an RFA system with suggested settings from 100° to 120°C, double cycling for the first vein segment, with one impulse for each additional vein segment.

Concurrent phlebectomy or foam sclerotherapy was used in most cases. Concomitant selective ligature or foam sclerotherapy of incompetent perforator veins (PVs) was also performed. Patients were discharged home with instructions to wear compression stockings for ≥10 days.

### Statistical analysis

The demographic and anatomic characteristics of the 1300 patients and procedure, postoperative, and follow-up data were collected in a dedicated database (Excel software; Microsoft). The collected data are presented as the mean ± standard deviation or frequencies and percentages and were compared using a two-tailed *t* test and the Pearson χ^2^ test. The GSV occlusion rate and freedom from reintervention were computed using the Kaplan-Meier method. The Kaplan-Meier curves are displayed up to a standard error of <0.10. A 95% confidence interval (CI) was used for all variables of the underlying distribution.

The primary analysis was not adjusted for covariates. A logistic regression model using stepwise selection was used to identify predictors of the different end points. Data were entered into the model if the univariate *P* value was < .05. Collinearity and overfitting were assessed using a stepwise regression model and the Pearson correlation test. On multivariate analysis, clinical factors or potential confounding variables are presented as odds ratios (ORs) with the 95% CIs. All analyses were performed using the RStudio package, version 0.99.902 (RStudio, Inc).

## Results

Between 2009 and 2019, 1568 limbs with incompetent GSV were treated in 1300 consecutive patients referred from two high-volume vein centers (San Raffaele Hospital, n = 748; San Camillo Hospital, n = 552), using the ClosureFast system. The demographic and anatomic characteristics of all patients are listed in Table I. The mean age was 53.5 ± 12.9 years, and most of the patients were women (72%). Of the 1300 patients, 246 (23%) were current smokers, 122 (11%) had diabetes mellitus, 18 (1.7%) had a history of DVT, and 20 (1.9%) had thrombophilia.

**Table I:** Preoperative clinical characteristics

Variable	Value
Demographic characteristics (n = 1300)	
Age, years	53 ± 12.9
Female sex	924 (71.9)
BMI >25 kg/m^2^	23 ± 2.3
Hypertension	404 (31.1)
DM	161 (12.3)
Smoker	301 (23)
Dysthyroidism	241 (18.5)
CAD	74 (5.7)
Pregnancy history	649 (45)
DVT history	25 (1.9)
Thrombophilia	23 (1.8)
Limbs treated (n = 1568)	
CEAP	
C1	74 (4.7)
C2	904 (57.6)
C3	468 (29.8)
C4	80 (5.1)
C5	16 (1.0)
C6	26 (1.6)
Right GSV	870 (55.5)
Right SSV	20 (1.2)
Right ASV	78 (4.5)
Left GSV	698 (44.5)
Left SSV	3 (0.2)
Left ASV	79 (5)
Perforator vein incompetence	373 (23.9)
ASV incompetence	154 (9.8)
CDUS preoperative characteristics	
GSV diameter at 5 cm from SFJ, mm	9.2 ± 2.9
GSV diameter at 10 cm from SFJ, mm	8.7 ± 2.6
GSV diameter at 20 cm from SFJ, mm	7.4 ± 2.1
Mean lesion length, cm	32.8 ± 6.0
ASV origin in SFJ	425 (27.2)
ASV origin from GSV	1143 (72.8)
Clinical score	
VAS score	56 (35-89)
VCSS	6 (4-8)

*ASV,* Accessory saphenous vein; *BMI,* body mass index; *CAD,* coronary artery disease; *CDUS,* color Doppler ultrasound; *CEAP,* clinical, etiology, anatomy, pathophysiology; *DM,* diabetes mellitus; *DVT,* deep vein thrombosis; *GSV,* great saphenous vein; *SFJ,* saphenofemoral junction; *SSV,* small saphenous vein; *VAS,* visual analog scale; *VCSS,* venous clinical severity score.

Data presented as mean ± standard deviation or median (interquartile range) for continuous variables or number (%) for absolute variables.

Most of the limbs were classified as CEAP C2 (57.6%) and C3 (29.8%), with a mean preoperative GSV diameter and lesion length of 8.2 ± 2.3 mm and 32.8 ± 6 cm, respectively. PVs were detected in 376 limbs (23.9%). An ASV was documented in 1512 limbs (96.4%), and its confluence was directly into the SFJ in 425 limbs (27.1%) and into the GSV in the other limbs. The ASV was incompetent in 154 patients (9.8%). The ASV was treated with RFA in 120 patients (77.9%) and with foam sclerotherapy in 34 patients (22.1%).

The technical success rate was 99.7%. No intraoperative complications were documented. Phlebectomy were performed concurrently in 1120 of 1568 limbs (71.5%). Foam sclerotherapy was used for 52 limbs (3.3%). For 392 limbs (25.2%), RFA was performed alone. One patient underwent phlebectomy 3 months after the initial procedure because of the persistence of varicose veins, incomplete symptom resolution reported by the patient, and a persistent cosmetic concern. All patients had 1 week of follow-up. Of the 1568 limbs, 50 (3.2%) developed ecchymosis, 32 (2.4%) had hyperpigmentation, and 22 (1.4%) developed phlebitis. Heat-induced thrombosis occurred in 0.9% of cases but no DVT or pulmonary embolism was reported. No statistically significant differences were found between those with and without recurrence, except for those who received phlebectomy or foam sclerotherapy (Table II). Comparing these subgroups of patients, the recurrences rate was higher for those who had received foam sclerotherapy than for those who had received phlebectomy (*P* < .001).

**Table II:** Intraoperative details and analysis of perioperative complications ≤1 week after intervention stratified by recurrence (n = 1300 patients)

Variable	Overall (n = 1568 limbs)	Recurrence		*P* value
		No (n = 1410)	Yes (n = 158)	
Intraoperative details				
Anesthesia				
Local	1363 (86.9)	735 (52.1)	86 (54.4)	.814^a^
Local and sedation	205 (13.1)	675 (48.9)	72 (45.6)	.798^a^
Intervention time, minutes	35 (30-43)	35 (30-43)	36 (30-41)	.646^b^
Phlebectomy	1120 (71.5)	1044 (74)	76 (48.1)	**.003**^a^
Foam sclerotherapy	52 (3.3)	39 (2.7)	13 (8.2)	**.001**^a^
RFA alone	396 (25.2)	327 (23.3)	69 (43.6)	**.001**^a^
PV ligation	202 (12.9)	176 (12.5)	26 (16.4)	.220^a^
PV foam sclerotherapy	174 (11.1)	120 (8.5)	54 (34.1)	**.001**^a^
No technical success	5 (0.3)	3 (0.2)	2 (1.26)	.141^a^
Postoperative details (≤1 week)				
Ecchymosis	50 (3.2)	46 (3.2)	4 (2.5)	.808^a^
Hyperpigmentation	32 (2.04)	26 (1.8)	6 (3.8)	.191^a^
Pain	38 (3.6)	32 (2.7)	6 (3.8)	.379^a^
Paresthesia	4 (0.4)	4 (0.3)	0 (0.0)	1.00^a^
Phlebitis	22 (1.4)	18 (1.27)	4 (2.5)	.371^a^
EHIT	14 (0.9)	10 (0.7)	4 (2.5)	.067^a^
DVT	0 (0)	0 (0.0)	0 (0.0)	–

*DVT,* Deep vein thrombosis; *EHIT,* endovenous heat-induced thrombosis; *RFA,* radiofrequency ablation; *PV,* perforator vein.

Data presented as number (%) or median (interquartile range).

Boldface *P* values represent statistical significance.

a *P* value determined using the χ^2^ test.

b *P* value determined using the Student *t* test.

Incompetent PVs were treated in 373 limbs (23.9%). Surgical ligation of the PVs was performed in 201 limbs (53.9%) and foam sclerotherapy in 172 limbs (46.1%). No differences were observed between the patients with and without recurrence (*P* = .220). However, the recurrences rate was higher after foam sclerotherapy than after surgical ligation (*P* = .001).

**Fig 1:**
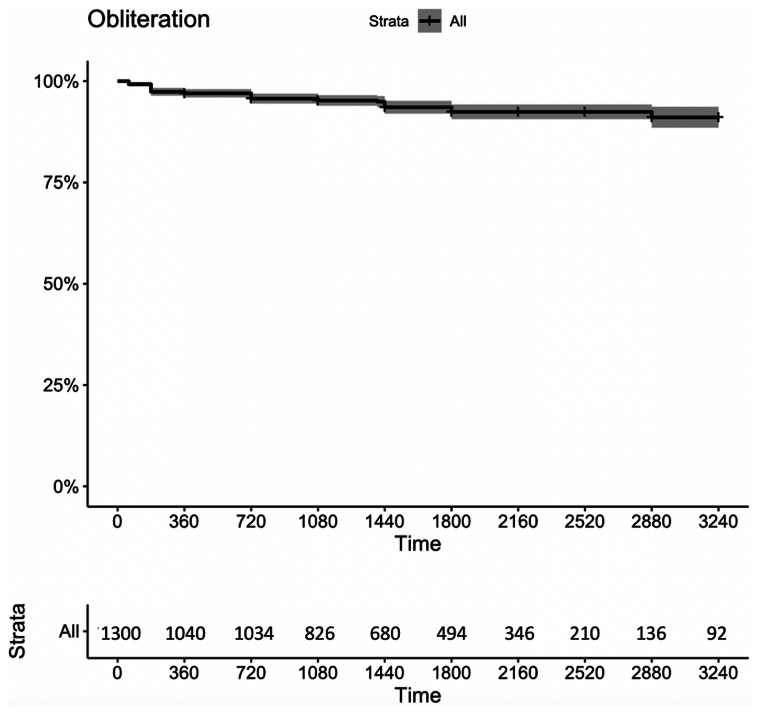
Kaplan-Meier survival curves with 95% confidence intervals (CIs) for great saphenous vein (GSV) obliteration rate.

**Fig 2:**
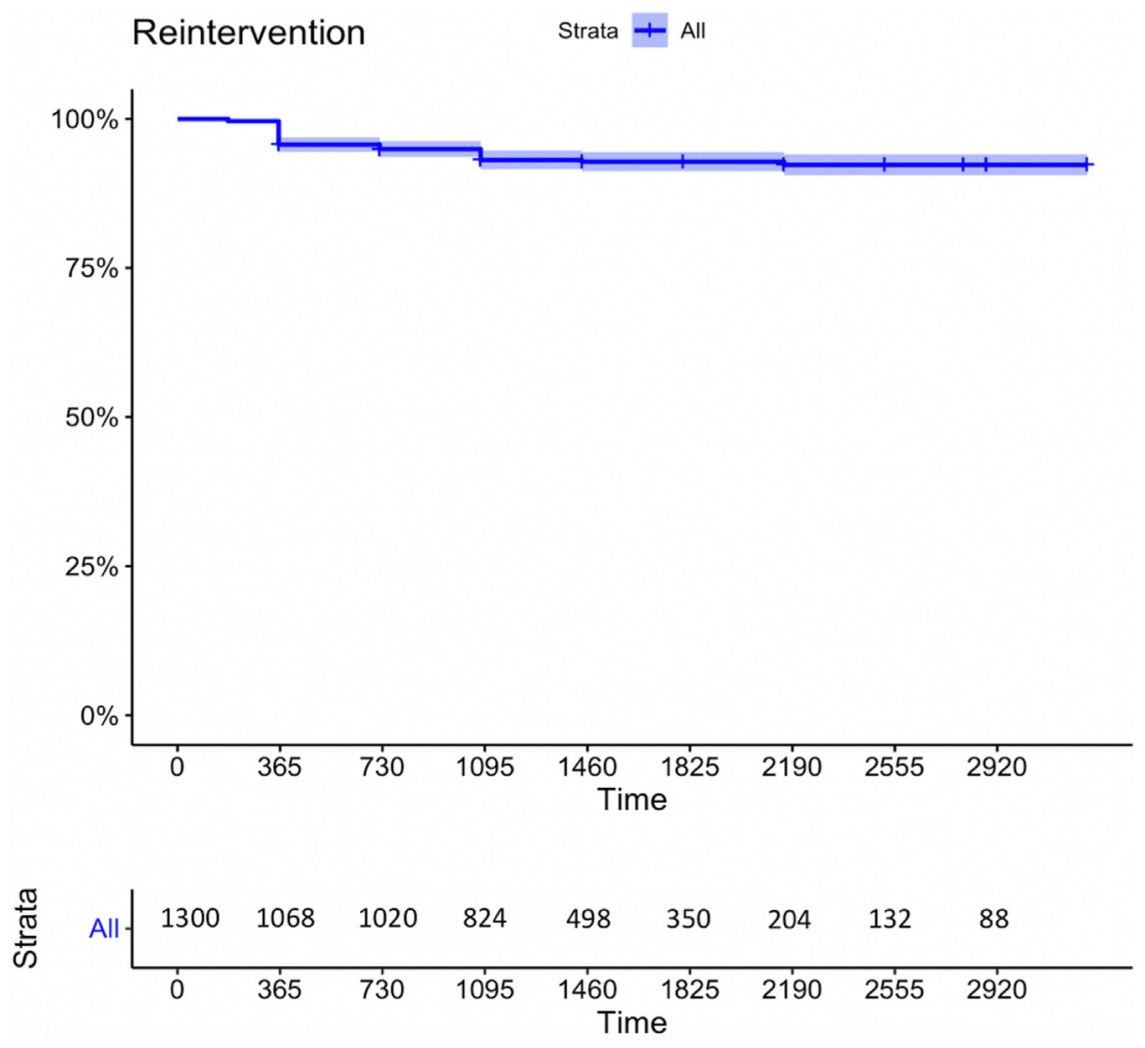
Kaplan-Meier survival curves with 95% confidence intervals (CIs) for reintervention rate.

At a mean follow-up of 57.2 ± 25.4 months, 232 patients were lost to follow-up and excluded from analysis. GSV occlusion was 100% for the patients with a 100% technical successful rate at ≤1 week after intervention. The occlusion and freedom from reintervention rates were 97% (95% CI, 96%-98%) and 95.7% (95% CI, 94.5%-96.9%) at 1 year, 95.2% (95% CI, 93.9%-96.5%) and 93.1% (95% CI, 91.5%-94.7%) at 3 years, and 92.4% (95% CI, 90.6%-94.2%) and 92.8% (95% CI, 91.2%-94.4%) at 5 years, respectively (Figs 1 and 2).

Of the 1568 limbs, 158 (10%) developed recurrence during the follow-up. Of these 158 patients, 32 (20.2%) had RVVs and GSV recanalization (group B1), 38 (24.1%) had GSV recanalization without RVVs (group B2), and 88 (55.7%) had RVVs without GSV recanalization (group B3). More patients in group B1 had a history of phlebitis (43.7%; *P* = .01) and deep vein incompetence (37.5%; *P* = .03) compared with the other groups. The analysis revealed that of 158 the limbs with recurrence in the territory of the GSV treated solely with ablation, 69 (43.6%) had developed new lesions. In contrast, 89 (56.4%) experienced recurrence when ablation of the GSV was associated with phlebectomy (n = 76) or foam sclerotherapy (n = 13), without statistically significant difference between groups (*P* = .458).

Of the limbs with recurrence, 82 (51.9%) were symptomatic, of which 78 (95%) underwent reintervention during follow-up (Fig 2). Of the 82 symptomatic limbs, 30 (36.6%) were in group B1 (GSV recanalization and RVVs), 14 (17.1%) were in group B2 (GSV recanalization without RVVs), and 38 (46.3%) were in group B3 (RVVs without GSV recanalization). PV incompetence was more frequent in group B3 than in the other groups (70.5%; *P* < .001; Table III).

**Table III:** Preoperative clinical characteristics for 158 patients with recurrence

Demographic characteristics	GSV recanalization		RVVs without GSV recanalization (n = 88)	*P* value
	With RVVs (n = 32)	Without RVVs (n = 38)		
Age, years	55 (43-76)	60 (48-65)	58 (48-68)	.451^a^
Female sex	24 (76%)	28 (74%)	66 (75%)	.991^b^
BMI >25 kg/m^2^	24 (23-25)	24 (22-26)	24 (22-26)	.713^a^
DM	8 (25)	6 (15.8)	14 (15.9)	.485^b^
Smoker	6 (18.7)	6 (15.8)	22 (25)	.473^b^
Dysthyroidism	4 (12.5)	10 (26.3)	16 (18.2)	.332^b^
CAD	10 (31.2)	10 (26.3)	14 (15.9)	.466^b^
VV history	16 (50)	20 (52.6)	40 (45.5)	.743^b^
DVT history	6 (18.7)	2 (5.3)	8 (9.1)	.165^b^
Phlebitis history	14 (43.7)	4 (10.5)	30 (34.1)	**.017**^b^
DV incompetence	12 (37.5)	4 (10.59	22 (25)	.018^b^
Thrombophilia	0 (0.0)	2 (5.3)	4 (4.5)	.449^b^
Preoperative GSV, mm				
Proximal diameter	10 (8-10)	8 (7-12)	8 (7-10)	.274^a^
Middle diameter	12 (8-14)	8 (7-12)	9 (7-11)	.362^a^
Distal diameter	10 (9-10)	7 (6-9)	8 (6-9)	.233^a^
Lesion length treated, cm	30 (28-30)	34 (30-35)	30 (28-33)	.122^a^
CEAP class				
C2	10 (31.2)	16 (42.1)	34 (38.6)	.642^b^
C3	6 (18.7)	16 (42.1)	20 (22.8)	**.040**^b^
C4	2 (6.2)	2 (5.3)	18 (20.4)	.190^b^
C5	4 (12.4)	2 (5.3)	6 (6.8)	.481^b^
C6	10 (31.2)	2 (5.3)	10 (11.4)	.059^b^
PV reflux	14 (43.7)	14 (36.8)	62 (70.45)	**.011**^b^

*BMI,* Body mass index; *CAD,* coronary artery disease; *CEAP,* clinical, etiology, anatomy, pathophysiology; *DM,* diabetes mellitus; *DV,* deep vein; *DVT,* deep vein thrombosis; *GSV,* great saphenous vein; *PV,* perforator vein; *RVVs,* recurrent varicose veins; *VV,* varicose vein.

Data presented as number (%) for continuous data or mean ± standard deviation or median (interquartile range) for categorical data.

Boldface *P* values represent statistical significance.

a *P* value determined using the Student *t* test.

b P value determined using the χ^2^ test.

At 1 year after intervention, 1068 patients were available for analysis (Table IV). Patients with recurrence (2.6%) had a higher body mass index (BMI; median, 25 kg/m^2^ [interquartile range (IQR), 24-26 kg/m^2^]; vs median, 23 kg/m^2^ [IQR, 22-24 kg/m^2^]; *P* < .001) and a higher rate of current smoking (33.3% vs 22.4%; *P* = .04) compared with the patients without recurrence. The mean GSV diameter was 4.2 ± 1.5 mm (with vs without recurrence, 4.1. ± 1.5 vs 2.9 ± 1.2; *P* < .01).

**Table IV:** Perioperative complication analysis stratified by recurrence during follow-up

Follow-up	Overall	Recurrence		*P* value^a^
		No	Yes	
At 1 year	1068 (100)	1040 (98)	28 (3)	NA
Ecchymosis	37 (3.5)	34 (3.2)	3 (10.5)	.138
Hyperpigmentation	45 (2.7)	43 (4.1)	2 (7)	.787
Pain	31 (2.9)	30 (2.8)	1 (3.5)	.717
Paresthesia	12 (1.1)	11 (1.0)	1 (3.5)	.749
Phlebitis	11 (1.1)	11 (1.0)	0 (0.0)	1.00
Edema	20 (1.9)	18 (1.7)	2 (7)	.191
DVT	0 (0.0)	0 (0.0)	0 (0.0)	NA
At 3 years	776 (100)	716 (93)	60 (8)	NA
Ecchymosis	26 (3.3)	24 (3.3)	2 (3.4)	.714
Hyperpigmentation	16 (2.1)	15 (2.1)	1 (1.7)	.799
Pain	14 (1.8)	12 (1.7)	2 (3.4)	.687
Paresthesia	8 (1.0)	8 (1.1)	0 (0.0)	1.00
Phlebitis	12 (1.5)	18 (2.5)	4 (6.8)	.659
Edema	12 (1.5)	10 (1.4)	2 (3.4)	.548
DVT	0 (0.0)	0 (0.0)	0 (0.0)	NA
At 5 years	450 (100)	380 (85)	70 (16)	NA
Ecchymosis	15 (3.3)	11 (2.9)	4 (5.7)	.424
Hyperpigmentation	12 (2.6)	9 (2.3)	3 (4.3)	.628
Pain	8 (1.8)	5 (1.3)	3 (4.3)	.232
Paresthesia	4 (0.9)	3 (0.8)	1 (1.4)	.862
Phlebitis	0 (0.0)	0 (0.0)	0 (0.0)	NA
Edema	9 (2)	7 (1.8.0)	2 (2.9)	.934
DVT	0 (0.0)	0 (0.0)	0 (0.0)	NA

*DVT,* Deep vein thrombosis; *NA,* not applicable.

a *P* value determined using the χ^2^ test.

At 3 years after the intervention, 776 patients completed the follow-up examinations (Table IV). Of the 776 patients, 60 had recurrence (7.7%). The latter patients were more affected by diabetes mellitus (20.1% vs 10.1%; *P* = .002) and incompetent PVs (58.2% vs 31.9%; *P* = .001). The mean GSV diameter was 2.7 ± 1.4 mm (with vs without recurrence, 2.8 ± 1.1 vs 1.8 ± 1.5; *P* < .001).

At 5 years after intervention, 450 patients were available for analysis (Table IV). The 70 patients with recurrence (15.5%) more frequently had hypertension (18.1% vs 7.1%; *P* = .003) and, similar to the 1- and 3-year follow-up data, had a higher rate of incompetent PVs (58.3% vs 34.1%; *P* < .001) compared with the patients without recurrence. The mean GSV diameter was 2.2 ± 0.9 mm (with vs without recurrence, 2.5 ± 1.3 vs 1.4 ± 1.2; *P* < .001).

On multivariate analysis, the direct confluence of the ASV into the SFJ (OR, 1.561; 95% CI, 1.0-7.04; *P* = .032), a history of pregnancy >2 (OR, 3.68; 95% CI, 1.19-11.36; *P* = .023), C4 (OR, 6.41; 95% CI, 1.36-30.28; *P* = .019), C5 (OR, 7.76; 95% CI, 1.15-52.25; *P* = .035), and preoperative GSV diameter >10 mm (OR, 1.82; 95% CI, 1.65-4.03; *P* = .043) were predictive factors for GSV recanalization (Table V). Moreover, age >70 years (OR, 1.04; 95% CI, 1.01-1.06; *P* = .014) and incompetent PVs (OR, 1.17; 95% CI, 0.65-2.03; *P* = .018) were also risk factors for RVVs (Table VI).

**Table V:** Univariate and multivariate analysis results identifying predictors of great saphenous vein (GSV) recanalization

Variable	Univariate		Multivariate	
	OR (95% CI)	*P* value	OR (95% CI)	*P* value
Age >70 years	5.82 (2.04-15.6)	**<.001**	–	NA
Diabetes	2.83 (0.93-7.64)	**.011**	–	NA
DVT history	2.60 (1.09-6.18)	**.003**	–	NA
Pregnancies >2	4.34 (2.12-13.40)	**<.001**	3.68 (1.19-11.36)	**.023**
GSV diameter >10 mm	5.03 (2.07-13.32)	**<.001**	1.82 (1.65-4.03)	**.043**
CEAP C4	6.62 (1.06-32.88)	**.003**	6.41 (1.36-30.28)	**.019**
CEAP C5	6.78 (2.06-50.23)	**<.001**	7.76 (1.15-52.25)	**.035**
Direct confluence of ASV in SFJ	3.37 (1.42-8.40)	**<.001**	1.56 (1.0-7.04)	.**032**

*ASV,* Accessory saphenous vein; *CEAP,* clinical, etiology, anatomy, pathophysiology; *CI,* confidence interval; *DVT,* deep vein thrombosis; *NA,* not applicable; *OR,* odds ratio; *SFJ,* saphenofemoral junction.

Boldface *P* values represent statistical significance.

**Table VI:** Univariate and multivariate analysis results identifying predictors of recurrence

Variable	Univariate		Multivariate	
	OR (95% CI)	*P* value	OR (95% CI)	*P* value
Age >70 years	4.19 (1. 56-15.6)	**<.001**	1.04 (1.01-1.06)	**.014**
DM	2.11 (0.71-5.37)	**.050**	–	NA
BMI >30 kg/m^2^	4.06 (0.52-22.72)	**.037**	–	NA
Pregnancies >2	2.75 (0.24-5.71)	**.043**	–	NA
GSV diameter >10 mm	1.34 (0.09-4.35)	**.046**	–	NA
CEAP 4	6.30 (1.16-30.74)	**.002**	13.3 (3.72-46.34)	**.001**
CEAP 5	13.84 (1.64-71.11)	**<.001**	11.11 (3.56-35.04)	**.001**
Perforator reflux	2.34 (1.12-5.23)	**.002**	1.17 (0.65-2.03)	**.018**

*BMI,* Body mass index; *CEAP,* clinical, etiology, anatomy, pathophysiology; *CI,* confidence interval; *DM,* diabetes mellitus; *GSV,* great saphenous vein; *NA,* not applicable; *OR,* odds ratio.

Boldface *P* values represent statistical significance.

**Fig 3:**
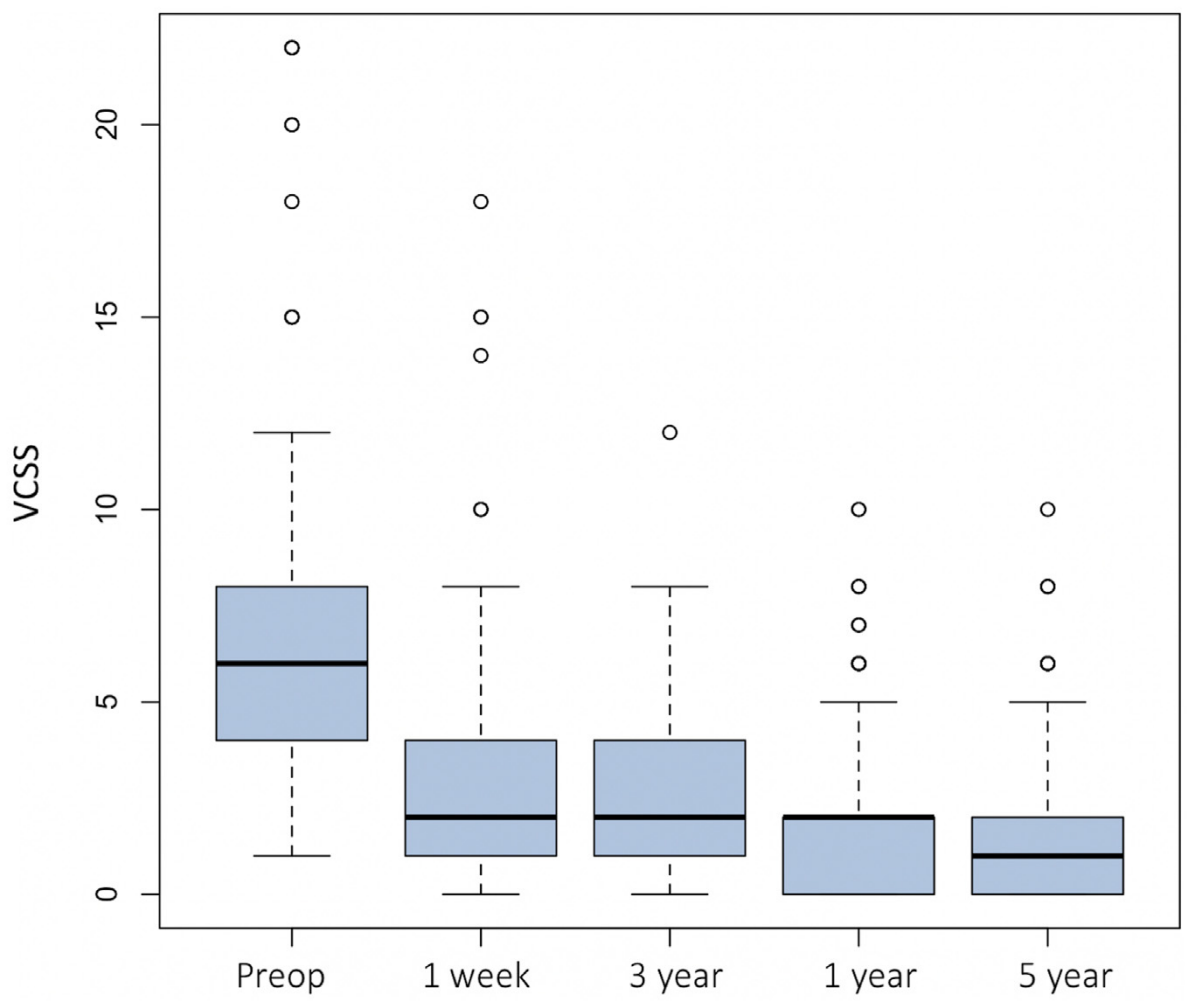
Venous clinical severity score (VCSS) before and after radiofrequency ablation (RFA) for great saphenous vein (GSV) incompetence. The median (*line within the box*) and range (*error bar*), excluding outliers (*circles*), are shown.

During follow-up, the VCSS constantly decreased in patients with and without recurrence. The VCSS had decreased a median of 4 points (IQR, 2.8-6 points) from baseline (*P* < .01). The VCSS was 4 (95% CI, 2-6) for those without recurrence and 3 (95% CI, 2-5) for those with recurrence (*P* ≤ .001; Fig 3).

## Discussion

Saphenofemoral HLS of the GSV has been the most common approach to manage truncal reflux in symptomatic patients. However, its use has been questioned and it has gradually been supplanted by endovenous procedures. Several studies have indicated that endothermal ablation results in a shorter postoperative hospital stay, a quicker return to work, and a low incidence of periprocedural complications,^2^^,^^9^, ^10^, ^11^ with confirmed safety and effectiveness and a low recanalization rate in the early and mid-term periods.^2^ Compared with HLS, endothermal ablation offers reduced invasiveness and better cost-effectiveness; thus, endothermal ablation has been recommended by guidelines worldwide. Despite multiple endeavors to make the procedure less invasive and more efficient, the short- and long-term RVV rates after GSV endovenous ablation remain comparable to those after HLS.^12^ Two systematic reviews and meta-analyses reported a different etiology for RVVs after the two procedures: neovascularization was the most common cause of recurrence after HLS and recanalization was more common after endovenous ablation.^12^^,^^13^ In the case of RVVs after RFA of the GSV, the most prevalent causes include recanalization of the treated vessel and disease progression in an untreated vessel.^5^ According to Brake et al,^13^ the sources of RVV can be classified as strategic mistakes (before surgery), technical errors (during surgery), disease progression, and, finally, neovascularization. Although neovascularization and recanalization seem to be different, both occur from progression of the vasa vasorum to become new vessels. This can occur in any venous segment.^14^^,^^15^ Neovascularization typically occurs after vein transection and reconnection by new vessels. Recanalization occurs when the vein wall has been damaged on its internal structure (eg, by thermal or mechanical action, sclerosant, or thrombus [ie, superficial vein thrombosis]), and new vessels grow within the treated vein. Each of these new intraluminal vessels will remodel into larger tubes with demonstrable venous flow. Some will eventually become larger and develop reflux.^2^

Some risk factors for recurrent disease include advanced age, female sex, prolonged standing, an increased BMI, and previous recurrent disease.^16^ Deep insufficiency in the proximal common femoral vein can also influence SFJ recurrence. A retrospective study reported that 27% of the patients who developed SFJ recurrence had preoperatively had distal external iliac vein and common femoral vein incompetence cranial from the SFJ.^17^^,^^18^ Concomitant incompetence of the ASV, or the anatomy of its confluence at the SFJ, could affect the recurrence rate.^8^^,^^12^^,^^19^^,^^20^ Venous obstruction or direct compression, secondary to DVT or pelvic pathology, respectively, also can contribute to recurrence.

Therefore, it is essential to identify disease patterns and the patients with a greater risk of GSV recanalization preoperatively and those with the possibility of developing reflux in an untreated segment such as the ASV. Moreover, it would be useful to determine the postoperative risk factors for truncal ablation failure to allow those with a higher risk of RVV recurrence to be more closely monitored.

The associations between GSV recanalization after endovenous ablation and risk factors are inadequately understood. Despite various reported studies, the results are discrepant and reliable data are lacking. Most studies concerning GSV recanalization and/or recurrence in the venous system only describe the preoperative risk factors through CDUS or clinical patterns without providing evidence.^4^^,^^5^^,^^12^

A recent literature review identified a high BMI, large GSV diameter, and higher chronic venous insufficiency grade as the most frequently described risk factors for early and long-term GSV recanalization.^14^ In 2016, Van der Velden et al^17^ documented that CEAP class and GSV diameter were the strongest predictors of recanalization at 1 year after endovenous procedures. These data were confirmed in another recent study^14^ in which the investigators demonstrated through multivariate examination that CEAP classes C4 and C5, a preoperative GSV diameter >6 mm, and a history of smoking were independent risk factors for GSV recanalization. Additionally, age >61 years and postoperative complications such as hyperpigmentation, edema, and paresthesia were found to be dependent risk factors.

Our analysis, conducted at two high-volume centers, of RFA ablation for truncal reflux performed by skilled operators with same devices confirmed the findings of the previous series.^14^^,^^17^ However, our data also showed that direct confluence of the ASV into the SFJ (OR, 1.561; 95% CI, 1.0-7.04; *P* = .032) is a risk factor for GSV recanalization, confirming our previous study reported in 2020 in which we analyzed data from a smaller population. Our data recognized age >70 years (OR, 1.04; 95% CI, 1.01-1.06; *P* = .014) and incompetent PVs (OR, 1.17; 95% CI, 0.65-2.03; *P* = .018) as risk factors for RVVs in the long term.

An earlier analysis of the available literature suggested that BMI and saphenous trunk diameter are the only two recognized variables that could influence both short- and long-term recanalization rates, although other aspects such as chronic venous insufficiency status, patient sex, target vein treatment length, and other elements should be considered.^14^ In our analysis, BMI did not affect the outcomes in the population studied, potentially because of the narrow baseline range (BMI, 23.6 ± 2.2 kg/m^2^) and because the patients with and without recurrence had similar BMIs throughout the follow-up period.

Limited information is available regarding the postoperative complications and the risk of ablated vein recanalization. A recent investigation using the Vascular Quality Initiative Varicose Vein Registry revealed that patients with a GSV >5 mm were more likely to experience postoperative difficulties (0.6% vs 0%; *P* = .027) and a partial recanalization rate (0.8% vs 0.3%; *P* = .001) at an average follow-up of 138.13 ± 176.85 days.^21^ These data were confirmed in our analysis, which showed that a GSV diameter >10 mm was a predictor for GSV recanalization and RVVs.

This study aimed to show the long-term results of RFA of the GSV and to identify risk factors for its recanalization and RVV onset during the follow-up period in a large population of patients with CVD and truncal reflux. This retrospective analysis included 1300 nonrandomized, real-world outpatients, and our findings highlight several interesting points confirming the safety and efficacy of RFA for patients with truncal reflux and chronic venous disorders. We identified a large preoperative GSV diameter, direct confluence of the ASV into the SFJ, >2 pregnancies, and CEAP C4 and C5 as variables that might increase the risk of GSV recanalization. Moreover, the data emerging from this series suggest that the best results in terms of avoiding recurrence were obtained when RFA was associated with the treatment of varicose veins or PVs. Further analysis revealed that patients undergoing phlebectomy had a lower recurrence rate than those treated with sclerotherapy. Although this study includes a fairly large number of patients, the main limitations were that it was not randomized, the retrospective study design, and no analysis of patients who participated in a prevention program or made lifestyle changes after the procedures.

## Conclusions

RFA is a safe technique to ablate the GSV with a low complication rate and acceptable durability during 5 years of follow-up. However, patients with a high CEAP class and those with direct confluence of the ASV at the SFJ experienced higher rates of long-term GSV recanalization and RVVs. Moreover, in the case of recanalization, it is important to evaluate the GSV diameter, the presence of direct confluence of the ASV into the SFJ, and the effect of recanalization on the patient's clinical and quality of life assessments.

## Author contributions

Conception and design: DB, VA, GM

Analysis and interpretation: VA, RL

Data collection: VA, AP, FBAV, RC

Writing the article: DB, VA, RL

Critical revision of the article: DB, VA, AP, FBAV, RC, GM

Final approval of the article: DB, VA, AP, FBAV, RL, RC, GM

Statistical analysis: VA, RL

Obtained funding: Not applicable

Overall responsibility: DB

## Disclosures

None.
